# Large hypomethylated blocks as a universal defining epigenetic alteration in human solid tumors

**DOI:** 10.1186/s13073-014-0061-y

**Published:** 2014-08-26

**Authors:** Winston Timp, Hector Corrada Bravo, Oliver G McDonald, Michael Goggins, Chris Umbricht, Martha Zeiger, Andrew P Feinberg, Rafael A Irizarry

**Affiliations:** Center for Epigenetics, Johns Hopkins University School of Medicine, Baltimore, MD USA; Department of Biomedical Engineering, Johns Hopkins University School of Medicine, Baltimore, MD USA; Department of Medicine, Johns Hopkins University School of Medicine, Baltimore, MD USA; Department of Pathology, Johns Hopkins University School of Medicine, Baltimore, MD USA; Center for Bioinformatics and Computational Biology, Department of Computer Science, University of Maryland, College Park, MD USA; Departments of Surgery and Molecular Biology & Genetics, Johns Hopkins University School of Medicine, Baltimore, MD USA; Molecular Biology & Genetics, Johns Hopkins University School of Medicine, Baltimore, MD USA; Department of Biostatistics, Johns Hopkins Bloomberg School of Public Health, Baltimore, MD USA; Department of Biostatistics and Computational Biology, Dana Farber Cancer Institute and Department of Biostatistics, Harvard School of Public Health, Boston, MA USA

## Abstract

**Background:**

One of the most provocative recent observations in cancer epigenetics is the discovery of large hypomethylated blocks, including single copy genes, in colorectal cancer, that correspond in location to heterochromatic LOCKs (large organized chromatin lysine-modifications) and LADs (lamin-associated domains).

**Methods:**

Here we performed a comprehensive genome-scale analysis of 10 breast, 28 colon, nine lung, 38 thyroid, 18 pancreas cancers, and five pancreas neuroendocrine tumors as well as matched normal tissue from most of these cases, as well as 51 premalignant lesions. We used a new statistical approach that allows the identification of large hypomethylated blocks on the Illumina HumanMethylation450 BeadChip platform.

**Results:**

We find that hypomethylated blocks are a universal feature of common solid human cancer, and that they occur at the earliest stage of premalignant tumors and progress through clinical stages of thyroid and colon cancer development. We also find that the disrupted CpG islands widely reported previously, including hypermethylated island bodies and hypomethylated shores, are enriched in hypomethylated blocks, with flattening of the methylation signal within and flanking the islands. Finally, we found that genes showing higher between individual gene expression variability are enriched within these hypomethylated blocks.

**Conclusion:**

Thus hypomethylated blocks appear to be a universal defining epigenetic alteration in human cancer, at least for common solid tumors.

**Electronic supplementary material:**

The online version of this article (doi:10.1186/s13073-014-0061-y) contains supplementary material, which is available to authorized users.

## Background

The original observation of altered DNA methylation in cancer was widespread hypomethylation affecting as many as one-third of single copy genes and arising at the earliest stages [1]. Later studies identified CpG island hypermethylation as well [2]. More recently large heterochromatin regions termed LOCKs were found to become euchromatic in cancer cell lines [3] and partially methylated domains in embryonic stem cell lines [4]. Recent whole genome bisulfite sequencing studies of human colorectal cancer showed that hypomethylation affects large genomic regions corresponding to chromatin regions (LOCKs) and nuclear organization (LADs), accounting for >95% of the DNA methylation change in cancer [5,6]. This manifests itself as an intersample as an erosion of the normal methylation profile (hence increase in local/sequence related variation). Other work has identified similar hypomethylated blocks in breast cancer cell lines, and found direct correlation to chromatin modifications in the same population, [7]. More recent work has even identified these blocks in medulloblastomas without obvious genetic drivers, underscoring the importance of this type of epigenetic change in cancer, [8]. Large-scale hypomethylated blocks have also been associated with Epstein-Barr virus-induced B-cell immortalization [9], neuronally expressed genes [10], epigenetic changes prior to morphological transformation [11] age-related drift in the pathogenesis of MDS and AML [12].

Here we present the first integrated whole genome analysis of six different tumor types. We examined breast, colon, lung, pancreas adenocarcinoma (ACA), pancreas neuroendocrine tumor (PNET), and thyroid cancer samples. For the breast, colon, pancreas, and thyroid we also examined early stage samples (Table 1). Using these data, we have been able to identify blocks of altered methylation occurring in all of these cancer samples. Furthermore, it appears that many of the commonly reported hypermethylated areas (CpG islands) found in cancer are a subset of these blocks of altered methylation - we found that CpGs islands with altered methylation in these cancer samples were enriched within blocks, whereas most outside of blocks have unaltered methylation. A similar finding was reported for colon cancer [6]. This suggests a large scale phenomenon of methylation dysregulation in cancer, rather than a specific targeting of methylation change at given sites. Furthermore, this dysregulation is occurring early in cancer - even samples taken at early stages of cancer development, and thought to be benign, have evidence of these methylation blocks. This suggests changes may be occurring even before full cancer development - hints of this already appear in the literature with cancer DMRs correlating with areas of age-related methylation drift [13].

**Table 1:** **Tissue samples analyzed in this study**

	**Normal**	**Hyperplastic**	**Adenoma**	**Cancer**	**Metastatic**
Breast	10	0	4	10	0
Colon	18	0	10	9	16
Lung	11	0	0	9	0
Pancreas NET	4	0	0	5	0
Pancreas ACA	8	0	6	18	0
Thyroid	12	10	21	24	4

The methylation changes within the blocks are progressive over time, showing a greater drift away from the normal profile as the cancer progresses.

## Methods

### DNA isolation

DNA was isolated from tissue samples using either the MasterPure DNA Purification Kit (Epicentre) or DNeasy Blood and Tissue Kit (Qiagen) according the manufacturer’s protocol.

### Microarray processing

Purity and quantity of DNA was measured using nanodrop spectroscopy.

A total of 500 ng of gDNA was bisulfite treated using the EZ-DNA Gold methylation kit (Zymo Research). The resulting bisulfite treated DNA was then subjected to the manufacture’s protocol for the Illumina Infinium HumanMethylation450 BeadChip Kit. The data are publically available from GEO repository GSE53051, processed data can be browsed at [14].

### Single CpG analysis

**Figure 1:**
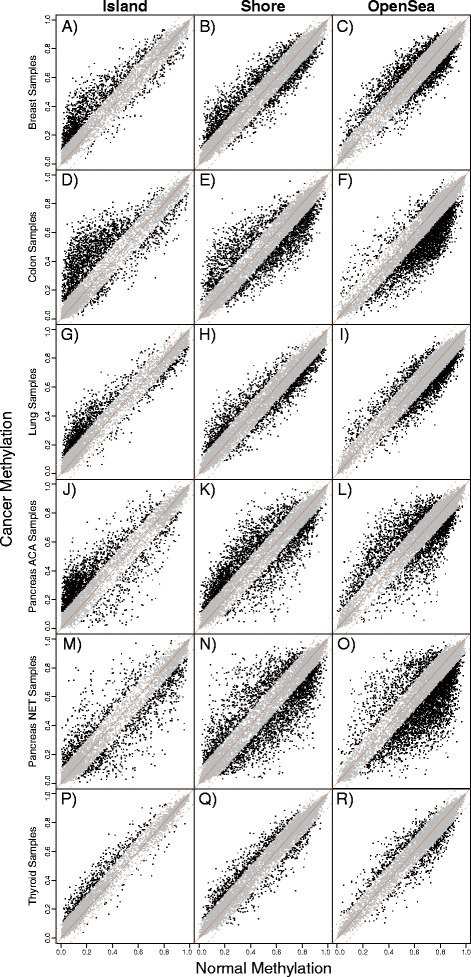
**Many of the methylation changes at single probes between cancer and normal are far from CpG islands. (A-C)** Scatter graph of individual probe average values in normal (x-axis) and cancer (y-axis) for island **(A)**, shore **(B)**, and open sea **(C)** probes. A random sampling of 10,000 probes is used for each region to illustrate the pattern. Panels **A-C, D-F, G-I, J-L, M-O, P-R** correspond to breast, colon, lung, pancreas adenocarcinoma, pancreas neuroendocrine tumor, and thyroid, respectively.

We preprocessed the Illumina HumanMethylation450 BeadChip methylation data using the Illumina default procedure implemented in the Bioconductor minfi package [15]. For the probe level analysis (Figure 1, Table 2) we averaged the values across all individuals within each category (for example, normal colon, breast cancer, thyroid adenoma, and so on) to produce one methylation profile for each. For each tissue, we computed the cancer, adenomas, and hyperplastic *versus* normal differences along with t-tests from which we obtained *P* values and then q values. For Table 2 we defined as significant probes showing a q value < 0.05 and with a difference magnitude larger than 0.1. The latter filter was performed to avoid including CpGs with differences not considered to be biologically meaningful. We re-ran our analysis with other cutoffs (data not shown) and the main conclusions did not change. For the analysis related to variance, we defined across individual standard deviation for each category as we did for the average values. R code for analysis is available upon request.

**Table 2:** **Percent of single CpGs that result in a q value <0.05 and effect size >0.10 when comparing cancer to normal samples with a t-test**

	**Colon**	**Lung**	**Pancreas NET**	**Pancreas ACA**	**Thyroid**	**Breast**
OpenSea-hypo	26	9.9	6.3	10	1.5	1.2
Shelf-hypo	22	8.7	5.6	7.6	1.5	1
Shore-hypo	13	6.2	3.7	7.5	0.94	1.1
Island-hypo	2.9	1.4	1.8	3	0.15	0.3
OpenSea-hyper	2	3.5	2.2	12	1.4	3.7
Shelf-hyper	2.1	2.5	2.3	11	0.87	2.7
Shore-hyper	8.2	4.7	2.4	14	1.5	4
Island-hyper	15	6.5	0.62	11	1.2	5

### Collapsed CpGs analysis

For each sample we collapsed measurements from islands, shores, and shelves into one value. Specifically, we averaged all the measurements within each of these regions to produce one measure per region. We then grouped any open sea probe that was within 500 bp from each other. If one of these regions exceeded 1,500 bp we broke them up into subsets. Details are available in the code of the cpgCollapse function in minfi [15]. This resulted in 223,497 collapsed regions: 26,571 CGIs, 47,344 CGI shores, 35,725 shelves, and 113,857 open sea. We then computed differences, standard deviations, and t-tests in the same way as we did for the single CpG analyses. R code for analysis is available upon request.

### Detecting blocks

**Figure 2:**
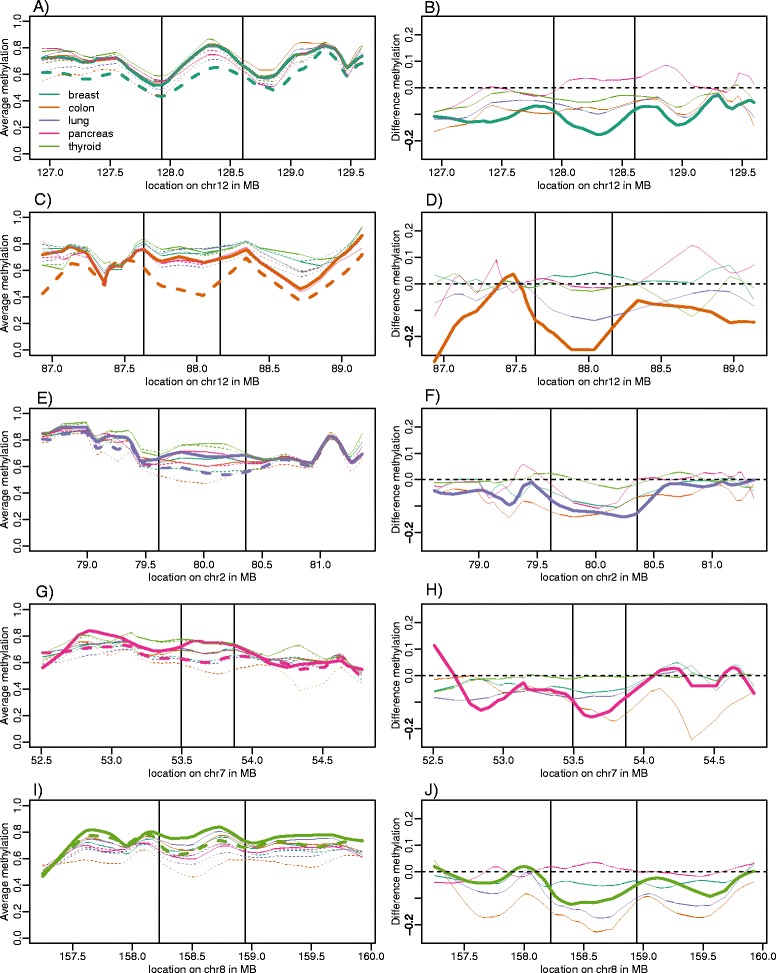
**Illustrative blocks of aberrant methylation in cancer.** Examples of a hypomethylated block. **(A)** Methylation values in normal and cancer samples for a block found in breast cancer, which is highlighted with a thicker line. **(B)** As (A) but showing difference between cancer and normal samples. The same plots are shown for colon **(C, D)**, lung **(E, F)**, pancreas **(G, H)**, and thyroid **(I, J)**.

To detect blocks (Figure 2) we used the method implemented in the blockFinder function in minfi [15]. Briefly, the 113,857 open sea collapsed values are split into regions. Then collapsed region that are within 250 kb from each other are grouped together. Finally the Bumphunter algorithm [16] is applied to detect regions exhibiting average differences between cases (for example, cancer samples) and controls (normal samples). To account for the large resolution character of blocks we loess-smoothed the data with a 250 kb window. Finally, a permutation test is run to determine which blocks have dimensions that are unlikely to occur by chance. We report blocks with q values <0.05 and containing at least five data points (collapsed regions). Note that this is an ad-hoc algorithm that does not use the standard definition of a *P* value [15]. Also note that the blockFinder algorithm reports candidate block regions that do not achieve these cutoffs. For the purposes of defining block and non-block regions we left these *gray area* regions outside of both categories. R code for analysis is available upon request.

### Block intersection *P* values

To calculate the *P* values shown in the caption of Table 3 we performed a Monte Carlo simulation. Specifically, for each list of blocks we created 1,000 equally sized lists of random blocks. In each of the random lists, each observed block in the original list had one region that was matched for genomic size (within 10% of the original size) and number of collapsed regions. For each region in the original list, we did this by sampling uniformly from all candidate genomic regions with the same number of collapsed regions and within 10% of the genomic width. Since the original list of observed blocks contains non-overlapping regions, we constrained the randomly generated block list to be non-overlapping as well (this was enforced by resampling until non-overlapping lists were created). With the 1,000 lists in place we computed the percent of regions in common with the colon blocks and kept these percentages to form the null distribution. *P* values were then calculated as the percent of values in the null distribution larger than the observed % agreement. All *P* values were less than 1/1,000 since none of the randomly created blocks had as many overlaps as the original lists. Note that this approach avoids biases due to the construction of the 450 K array since it uses matched regions that contain probes.

**Table 3:** **Large blocks of aberrant methylation identified in normal versus hyperplastic, adenoma, or cancer samples**

	**Blocks (n)**	**Total Mb inside blocks**	**Intersection with colon blocks (%)**	**25% ** **length quartile (Mb)**	**50% ** **length quartile (Mb)**	**75% ** **length quartile (Mb)**	**Median diff value**	**Hypo-meth. (%)**
Breast (Cancer-normal)	150	42.87	92	0.15	0.2	0.36	−0.097	83
Breast (DCIS-normal)	349	77.94	91	0.11	0.17	0.28	−0.078	79
Colon (Cancer-normal)	1889	746.93	100	0.16	0.28	0.53	−0.11	100
Colon (Adenoma-normal)	1917	576.63	79	0.13	0.22	0.38	−0.068	100
Lung (Cancer-normal)	702	217.72	87	0.14	0.23	0.38	−0.088	98
Pancreas (ACA-normal)	1114	183	53	0.087	0.14	0.2	−0.061	52
Pancreas (IPMN-normal)	1349	274.09	55	0.099	0.16	0.26	−0.09	99
Pancreas (NET-normal)	683	136.9	69	0.0955	0.16	0.25	−0.16	98
Thyroid (Cancer-normal)	351	63.25	82	0.091	0.15	0.235	−0.054	78
Thyroid (Adenoma-normal)	266	49.9	90	0.09275	0.15	0.24	−0.049	77

The ‘Blocks (n)’ column reports the total number of regions classified as blocks by our algorithm. ‘Total Mb inside blocks’ is the total number of megabases contained by these blocks. The ‘Intersection with colon blocks (%)’ represents a comparison of the hypomethylated blocks for the comparison represented by the row, to the hypomethylated blocks identified between colon normal and cancer samples; note that 100% of colon blocks are inside colon blocks. We tested if overlaps this extreme can be due to chance and found *P* values <0.001 (0 occurrences in 1,000 permutations). The next three columns are the 25th, 50th, and 75th percentiles of block region sizes. The ‘Median diff value’ is the median of the average difference between cases and controls are all regions defined as blocks. The ‘Hypo-meth. (%)’ column shows the percent of blocks that are hypomethylated.

### Gene expression hyper-variability analysis

We obtained frma [17] -normalized Affymetrix HGU133plus2 gene expression data for colon, breast, lung, pancreas, and thyroid tumors (curation and preprocessing of these data were previously described in [18]. We calculated the log ratio of observed to expected variability as described in Alemu et al. [19]. This method, which fits a local-likelihood regression method to estimate expected variability as a function of each gene’s mean expression level was shown to better control for variability of lowly-expressed genes than the commonly used coefficient of variation. To calculate enrichment in hypo-methylation domains we only considered probesets of genes with transcription start sites within the collapsed 450 k regions (described above) since these are the genomic regions covered by the 450 k array within which blocks can be detected [15].

### Profiles around CpG islands

**Figure 3:**
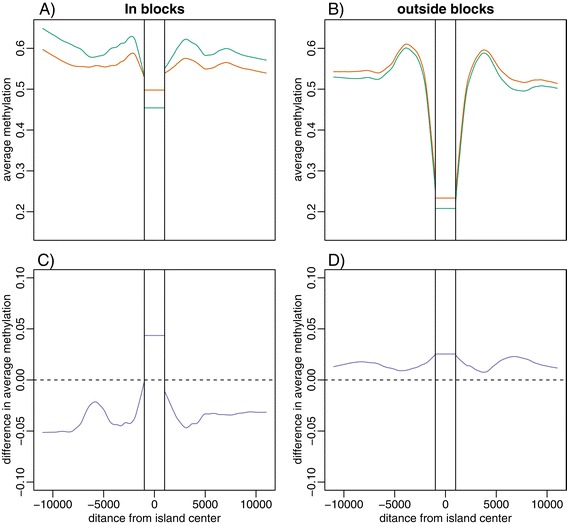
**Methylation changes in islands are enriched in the blocks. (A, B)** Average methylation in normal breast (green) *vs.* breast cancer (orange) samples plotted against distance from CpG islands both inside **(A)** and outside **(B)** of blocks. **(C, D)** Difference between cancer and normal sample methylation plotted against distance from CpG islands both inside **(C)** and outside **(D)** of blocks.

To study the profile surrounding CpG islands (Figure 3) we averaged across islands in the following way. We first obtained across-individual averages for each probe on each tissue category. We then separated islands into those inside blocks and those outside. For every CpG island we saved the value m of every probe within 10 kb and stored the distance d giving us several pairs (d,m) for each island. These were aggregated across all islands in consideration and a loess line was fit to these data. Then the average was computed for each island by aggregating all the values falling inside the CpG island. We did this analysis for islands inside and outside (not in grey area) hypomethylated. R code for analysis is available upon request.

## Results

### At the single CpG level many cancer to normal differences are far from CpG islands

We used the Illumina HumanMethylation450 BeadChip methylation array to probe cancer methylation 10 breast, 28 colon, nine lung, 38 thyroid, 18 pancreas cancers, and five pancreas neuroendocrine tumors as well as matched normal tissue from most of these cases and 51 premalignant lesions (Table 1). We stratified the 485,512 probes included in the array into CpG islands, CpG island shores (1 to 2,000 bp from island), CpG island shelves (2,001to 4,000 bp from island) and CpG open seas (>4,000 bp from island). For each tissue we computed cancer and normal across-individual averages for each probe. We then examined the differences between these pairs and declared a difference statistically and biologically significant when the q value was below 0.05 and the observed difference above 0.10 or below −0.10. We found that the majority were either hypomethylated probes located in open sea sites or hypermethylated CpG island probes (Table 2). For colon, lung, thyroid, and PNET there were more significantly hypomethylated probes than hypermethylated probes and for pancreas adenocarcinoma it was about the same. For breast there were more significantly hypermethylated probes than hypomethylated probes. In general, the hypomethylated probes were characterized by average methylation of approximately 75% in normal samples that dropped to approximately 60% in cancer samples (Figure 1). In contrast, the hypermethylated CpG island probes were characterized by approximately 10% methylation values for the normal samples increasing to approximately 40% in cancer (Figure 1). In both cases the methylation pattern moved from the extremes to the middle. The probes in CpG island shores are a hybrid of the other two types.

We computed the same summaries for the difference between early neoplastic tissue and normal tissue - specifically breast ductal carcinoma *in situ* (DCIS) and normal breast tissue, colon tubular adenoma and colon normal, intraductal papillary mucinous neoplasms (IPMNs) and normal pancreas, and follicular thyroid adenomas, and normal thyroid tissue. We observed the same trend of methylation changes in these early neoplasms as in the fully developed cancers (Additional file 1: Figure S1).

### Hypomethylated blocks are present in six cancer types

To determine if the observed hypomethylation is related to large hypomethylated blocks, previously identified for colon cancer using whole-genome bisulfite sequencing [5,6], we applied a new method that permits the detection of large differentially methylated regions using 450 k Methylation microarray data [15] (see Methods Section). To declare a region statistically and biologically significant, or blocks, we required a q value <0.05 and inclusion of at least five measurements (See methods). We also excluded the X and Y chromosomes. In the majority of cases, these blocks were hypomethylated regions, with median length on the order of hundreds of kb (Table 3); a full tabulation of identified blocks is included as Additional file 2: Data 1–11. Hypomethylated blocks were observed in each of the six cancer types as well as in the early stage samples (Table 3). Typically, blocks had an average methylation of approximately 75% in all the normal tissues (Figure 2A; solid lines), but in cancer became distinctly hypomethylated (Figure 2A; dotted liens). The difference between cancer and normal samples varied between types, with colon cancer showing the greatest area difference, and thyroid showing the least (Figure 2B). The great majority of detected blocks were hypomethylated (83%, 99% 98%, 99%, and 78% for breast, colon, lung, PNET, and thyroid, respectively) except for pancreas adenocarcinoma for which 48% were hypermethylated. For each hypomethylated block, we determined if it intersected with a colon hypomethylated block (at least 5,000 bps in common) and found these were highly co-localized (Table 3). This co-localization is observed in the top ranked blocks for each tissue type (Figure 2).

### For colon, lung, and breast hypermethylated islands are enriched inside blocks

We calculated the methylation distribution for cancer samples and normal samples and noted that while the distribution changed dramatically within hypomethylated blocks, it remained about the same outside blocks (Additional file 3: Figure S2). The methylation distributions in normal tissues were bimodal, with a peak near 10% -- primarily unmethylated CpG islands, and a peak at around 80% methylated (Additional file 3: Figure S2). In contrast, inside of blocks, the normal tissue was characterized by a unimodal distribution centered approximately 80% methylated, while cancer methylation is hypomethylated with different distributions for the different samples (Additional file 3: Figure S2). This was confirmed by studying the location of CpG islands showing statistically significant differences, with effect sizes surpassing 0.10. Samples that had a high level of hypermethylated islands, specifically, breast, colon, and lung, showed strong enrichment of hypermethylated islands within blocks (Table 4).

**Table 4:** **Hypermethylated CpG island location relative to blocks**

**Cancer type**	**CGIs in testable area (n)**	**Testable CGIs significantly (q <0.05; deltaM >0.1) hypermethylated (%)**	**CGIs in blocks (n)**	**Hypermethylated CGIs in blocks (%)**	**Odds ratio of CGI being in block and hypermethylated**	***P*** **value (Chi-squared test)**
Breast	24,206	11	316	20	2.5	<0.0001
Colon	19,827	19	3,769	30	4.1	<0.0001
Lung	22,661	8	1,310	12	2	<0.0001

The ‘CGIs in testable area (n)’ column shows the number of CpGs that are included in the analysis for that tissue. Note that CpGs that were inside region that were borderline from being called blocks are not included (see Methods for details). The next column shows the percentage of these CpG that were statistically significantly hypermethylated. The fourth column shows the number of the CGIs that were inside hypomethylated blocks. The next column shows the % that were hypermethylated among the CGIs that were in blocks (fourth column). The odds ratio and *P* value shown in the final two columns are based on the two by two table represented in each row: each CGI can be either hypermehtylated or not and inside a block or not.

### Inside blocks, methylation profiles flatten around CpG islands

We divided CpG islands into those inside and outside hypomethylated blocks. For each cancer type, for distances ranging from 1 bp to 15,000 bp in both genomic directions, we then computed the average methylation value across all islands for normal and cancer. We also computed this average for probes within CpG islands. We found that across all examined tissues these average methylation profiles went from a pattern of methylated outside islands to unmethylated inside islands back to methylated outside islands in normal tissues (Figure 3; Additional file 4: Figure S3). Outside blocks this pattern remained about the same for cancer samples, but within blocks the island methylation went up while the methylation right outside went down; both going from extreme to middle. The general trend is one of hypermethylation in islands, and hypomethylation of the surrounding area (Figure 3; Additional file 4: Figure S3).

### Hyper-variably expressed genes are enriched inside blocks

Gene expression hyper-variability in colon cancer was reported to be enriched in long hypomethylation blocks obtained from whole genome bisulfite sequencing [5]. To establish how consistent this association is across solid tumor types, we performed a similar association test for the five tissues profiled here. We obtained publicly available gene expression microarray data for tumors in each of the five tumors from the Gene Expression Barcode project [20,21]. Since expression is not available for normal samples in all tissues in this platform, we defined hyper-variability by calculating the log-ratio of observed variability to expected variability (conditioned on mean expression level) across tumor samples for each gene [19], and then tested association between hyper-variability (observed is twice the expected variability) and the gene’s TSS being inside a hypo-methylation block in each cancer type. We found that hyper-variability is enriched in the hypomethylation blocks in each cancer type (*P* <0.05) except breast cancer (*P* = 0.5) where the small number of hypomethylation domains results in lack of power. We also observed that the odds ratio for hypomethylation domain presence increases along with hyper-variability for all tissues (Additional file 5: Figure S4).

### The blocks occur in early neoplasms

**Figure 4:**
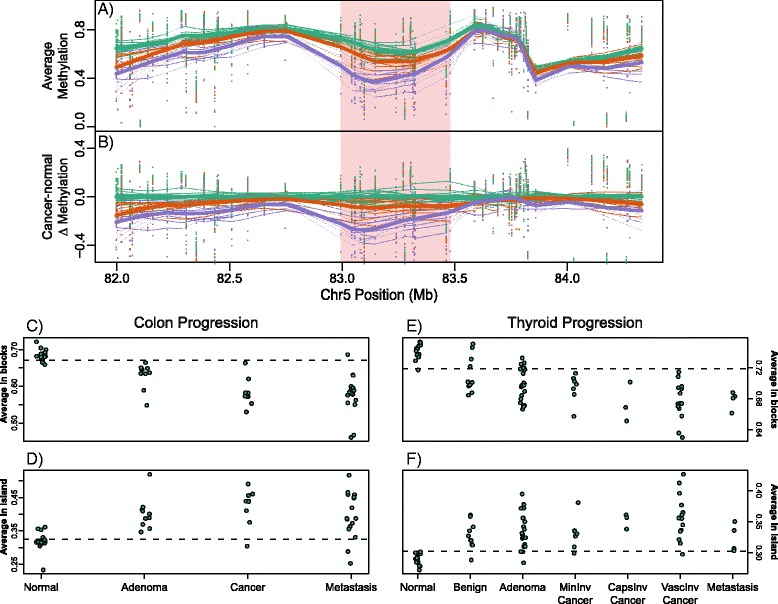
**Hypomethylated blocks occur early in cancer and increase with progression. (A)** Across-individual methylation averages for colon normal (green), adenoma (orange), and cancer (purple). The hypomethylated block in cancer is shown in pink. The points represent sample-specific values. Thick lines represent the sample type average, thin lines are each sample. **(B)** As (A) but the difference from the average normal. **(C, D)** Summarized average methylation values for blocks **(C)** and islands **(D)** in colon normal, adenoma, carcinoma, and metastasis samples. **(E, F)** Summarized average methylation values for blocks **(E)** and islands **(F)** in thyroid normal, benign hyperplastic (adenomatoid nodules), adenoma, minimally invasive carcinoma, capsular invasive carcinoma, vascular invasive carcinoma, and metastatic samples.

We examined several precursor lesions, including 10 colonic tubular adenomas, six pancreatic intraductal mucinous neoplasms (IPMNs), four breast ductal carcinoma *in situ* (DCIS), and 21 thyroid follicular adenomas. We found large numbers of hypomethylated blocks present even in these early neoplastic lesions, 1,880 blocks (23% of covered area) in colon tubular adenoma, 1,642 blocks (15% of covered area) in pancreas IPMNs, 327 blocks (3.7% of covered area) in breast DCIS, and 145 blocks (1% of covered area) in thyroid follicular adenomas. An example of this is plotted in Figure 4A - an 800 kbp block shows a progressive hypomethylation from normal colon tissue, to tubular adenoma, through to colon adenocarcinoma.

We wanted to further stratify the different stages of cancer to determine number, size, and magnitude of blocks during cancer progression. We divided the thyroid samples into benign thyroid lesions, thyroid follicular adenomas, minimally invasive thyroid carcinoma, capsular invasive thyroid carcinoma, vascular invasive thyroid carcinoma, and metastatic thyroid cancer and detected large numbers of blocks (Table 5). The number of blocks and the magnitude of changes increase in later stage cancers, but surprisingly we detected 51 blocks in benign hyperplastic adenomatoid nodules, which are not thought to have a pro-malignant potential [22]. Note that there may be even more dysregulated regions with methylation alteration too small to detect above noise in these samples.

**Table 5:** **Large blocks of aberrant methylation arise early in carcinogenesis and develop along with cancer**

	**Blocks (n)**	**Total Mb inside blocks**	**Intersection with colon blocks (%)**	**25% ** **length quartile (Mb)**	**50% ** **length quartile (Mb)**	**75% ** **length quartile (Mb)**	**Median diff value**	**Hypo. (%)**
Benign	142	25.3	90	0.098	0.14	0.21	−0.047	58
Follicular adenoma	270	50.17	90	0.08825	0.15	0.24	−0.048	77
Minimally invasive carcinoma	297	56.3	79	0.098	0.15	0.24	−0.054	94
Capsular invasive carcinoma	599	109.37	80	0.09	0.15	0.23	−0.074	90
Vascular invasive carcinoma	376	67.85	82	0.091	0.15	0.24	−0.053	78
Metastatic cancer	729	125.42	67	0.088	0.14	0.22	−0.066	92

Columns are as in Table 3. As in Table 3, *P* values were all below 0.001.

To summarize and evaluate how average methylation in blocks changes with progression (Figure 4A-B); we calculated a value for each sample using the average methylation level inside all blocks and inside all islands. Each sample then had a single value for blocks and a single value for islands. We performed this analysis for colon (Figure 4C-D) and thyroid (Figure 4E-F) with increasing stages of progression plotted along the x-axis. The normal samples in both cases had a clear tight clustering. However, even the earliest lesions showed marked alterations of large domains as seen in the later cancers.

## Discussion

There are three major results of this study. First, we have found that large hypomethylated blocks in cancer, which we first described in three colorectal cancers, are a universal feature of solid tumors. Blocks were found in all five tumor types, and in every cancer within them and hyper-variably expressed genes are enriched within hypomethylated blocks in all tumor types. Second, the hypomethylated blocks occur early in cancer: all four groups of premalignant lesions also showed the hypomethylated blocks. Thus more than any other mutation, copy number change, or individual methylation change, hypomethylated blocks represent the genetic signature of human solid tumors.

Third, in breast, colon, and lung cancer, altered DNA methylation in CpG islands are enriched in hypomethylated blocks. The hypermethylated islands contained in the blocks do not show hypermethylation *per se*, but flattening, that is, hypermethylation of the islands, and hypomethylation of the shores and shelves that flank them. Note that we may be underestimating the enrichment. First, we may be underestimating the genomic coverage of the blocks due to the statistically conservative threshold we use for defining them and because the array does not cover the entire genome (approximately two-thirds of the genome). Second, is the somewhat arbitrary choice of effect-size we used to define a hypermethylated CpG islands.

Note that these large domains defined by the hypomethylated blocks in cancer have been previously shown [5,6] to co-localize with regions showing heterochromatin modifications such as H3K9Me2 or H3K9Me3 (LOCKs) [3] or lamin-associated domains (LADs) [23] in normal cells. A recent report on epithelial-mesenchymal transition (EMT) showed that the loss of LOCKs is associated with this process reversibly, and the properties of cell spreading and chemoresistance can be inhibited by biochemical modification of LOCK demethylation [24]. In the original report of LOCKs, their loss was also described in cancer cell lines [3]. A recent report in prostate cancer demonstrates both hypo- and hypermethylation associated with reduced chromatin acetylation [25]. These results motivate a relatively new view of cancer epigenetics in which large-scale heterochromatin structures are disrupted generally, at least in solid tumors, leading to loss of both epigenetic and gene expression regulation, resulting in hyper-variability of gene expression [5]. These changes could even have interaction with large scale genetic domains important in cancer [26].

The data in this paper also offer a new perspective of the role of CpG island methylation in cancer. While historically the focus was on island hypermethylation, we see that: (1) much of the methylation change in cancer involves hypomethylated blocks; (2) many of the methylation changes at islands are more a flattening out of methylation rather than simply hypermethylation. The presence of these regions within the block domains suggests that the mechanism for island disruption is not necessarily island-specific but could be part of the loss of structural integrity of heterochromatin in these regions. That would explain the lack of data for specific mutations at islands or of island modifying or recognizing genes in most solid tumors. It is intriguing to speculate that the blocks might be the functional target of many of the chromatin modifiers already known to be disrupted in cancer. In particular, the advent of histone lysine demethylase therapy [27] seems particularly relevant to these structures [24].

## Conclusions

In summary, this is the first genome-scale analysis of DNA methylation in a large number of cancers and matched tissues, spanning six tumor types, and including premalignant lesions from four of the tumor types. This analysis allowed us to identify common features of the cancer epigenome in solid tumors and assess the timing of those changes. We also took advantage of new software that leverages the power of statistical smoothing and resampling to detect large statistically significant regions that are differentially methylated.

### Ethics and consent

Cryogenically stored freshly frozen samples were obtained from the Cooperative Human Tissue Network (National Cancer Institute (NCI)), and Johns Hopkins Hospital under an institutional review board–approved waiver of consent. This conforms to the Helsinki Declaration as well as local legislation.

## Additional files

Additional file 1: Figure S1. As in main Figure 1 but for premalignant lesions. (A-D) Scatter graph of individual probe average values in normal (x-axis) and cancer (y-axis) for islands (left), shore (middle), and open sea (right) probes. A, B, C, and D correspond to breast DCIS, colon tubular adenoma, pancreas IPMNs, and thyroid follicular adenomas, respectively. Additional file 2:
**Data 1–11.** Listing of individual blocks identified in comparisons between sample datasets. Columns in order are: chromosome, start coordinate of block, end coordinate of block, difference of average probe methylation per set within block, area (block length X difference of average probe methylation per set within block), *P* value for block difference significance, family-wise error rate of block difference, *P* value area and family-wise error rate area. Datasets are determined by comparisons between: (1) breast cancer *vs.* breast normal, (2) breast ductal carcinoma *in situ vs.* breast normal, (3) colon cancer *vs.* colon normal, (4) colon tubular adenoma *vs.* colon normal, (5) lung cancer *vs.* lung normal, (6) pancreas adenocarcinoma *vs.* pancreas normal, (7) pancreas intraductal papillary mucinous neoplasm *vs.* pancreas normal, (8) Pancreas neuroendocrine tumor *vs.* pancreas normal, (9) thyroid cancer *vs.* thyroid normal, (10) thyroid follicular adenoma *vs.* thyroid normal, (11) thyroid adenomatoid nodule (benign) *vs.* thyroid normal. Additional file 3: Figure S2. Methylation density plot for normal (green solid lines) and cancer samples (orange dotted lines) for (left) all CpGs, (middle) CpGs outside of blocks and (right) CpGs inside blocks. A, B, C, D, E, and F correspond to breast, colon, lung, pancreas adenocarcinoma, pancreas neuroendocrine tumor, and thyroid, respectively. Additional file 4: Figure S3. As Figure 3 but for (A) colon, (B) lung, (C) pancreas, and (D) thyroid. Additional file 5: Figure S4. Cancer gene expression hyper-variability is enriched in large hypomethylation domains in most solid tumor types. We obtained publicly available gene expression microarray data for each of the five tissues profiled and computed the log ratio of observed variability to expected variability (conditioned on mean expression) for each gene in each of the five cancer types. We plot the odds ratio of a gene's TSS being located within a detected hypomethylation block in each tissue given that observed to expected variability (OEV) is above increasing thresholds. We observed consistent increase in the odds ratio as the OEV threshold increases suggesting that gene expression hyper-variability is enriched in each tissue’s hypomethylation blocks.
