# Supplementary figures and images for: Large hypomethylated blocks as a universal defining epigenetic alteration in human solid tumors
Source: Genome Med. 2014 Aug 26;6(8):61. doi: 10.1186/s13073-014-0061-y (PMC4154522; doi:10.1186/s13073-014-0061-y)

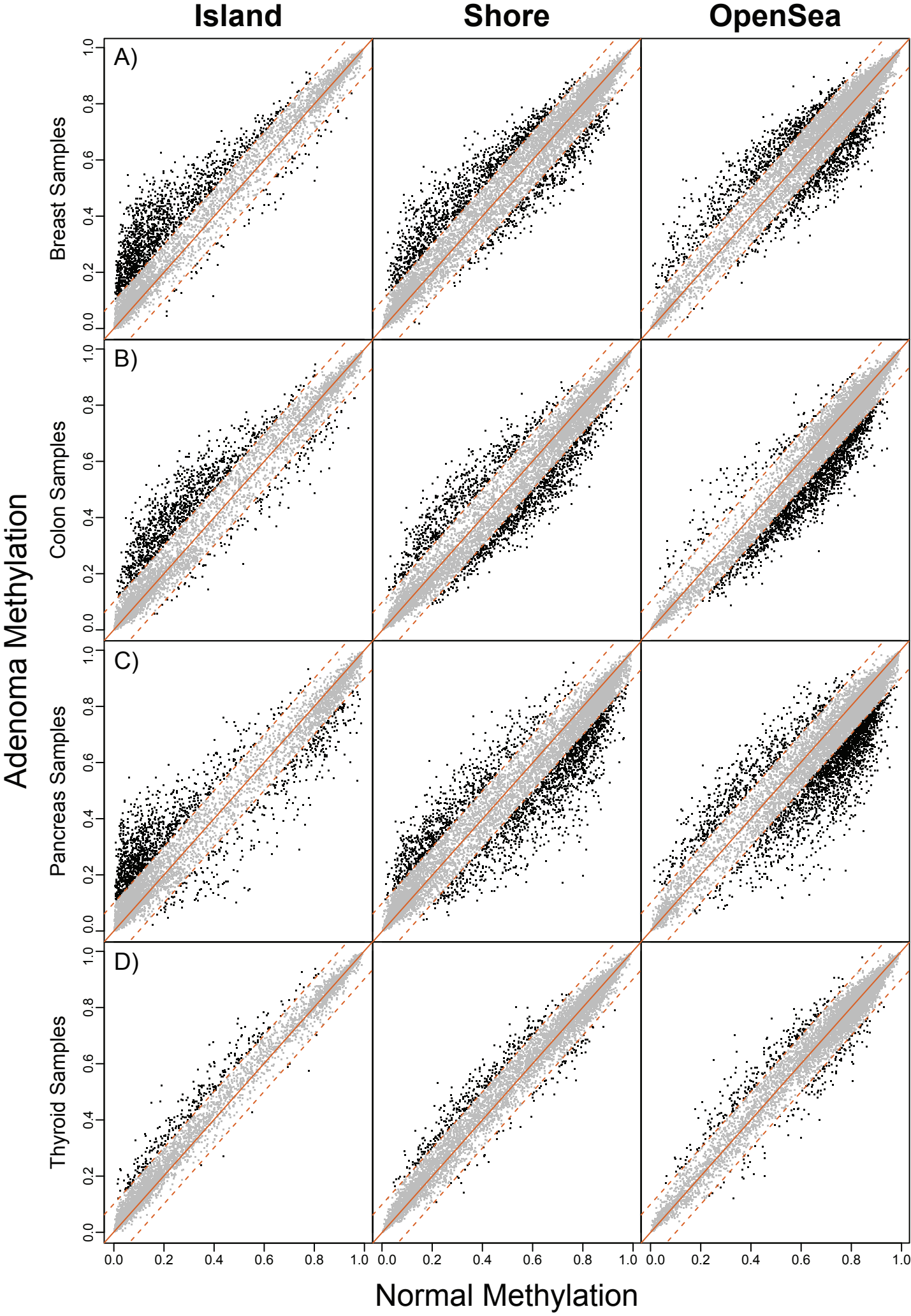

Supplement: Additional file 1: Figure S1. — As in main Figure 1 but for premalignant lesions. (A-D) Scatter graph of individual probe average values in normal (x-axis) and cancer (y-axis) for islands (left), shore (middle), and open sea (right) probes. A, B, C, and D correspond to breast DCIS, colon tubular adenoma, pancreas IPMNs, and thyroid follicular adenomas, respectively. [file 13073_2014_61_MOESM1_ESM.pdf]

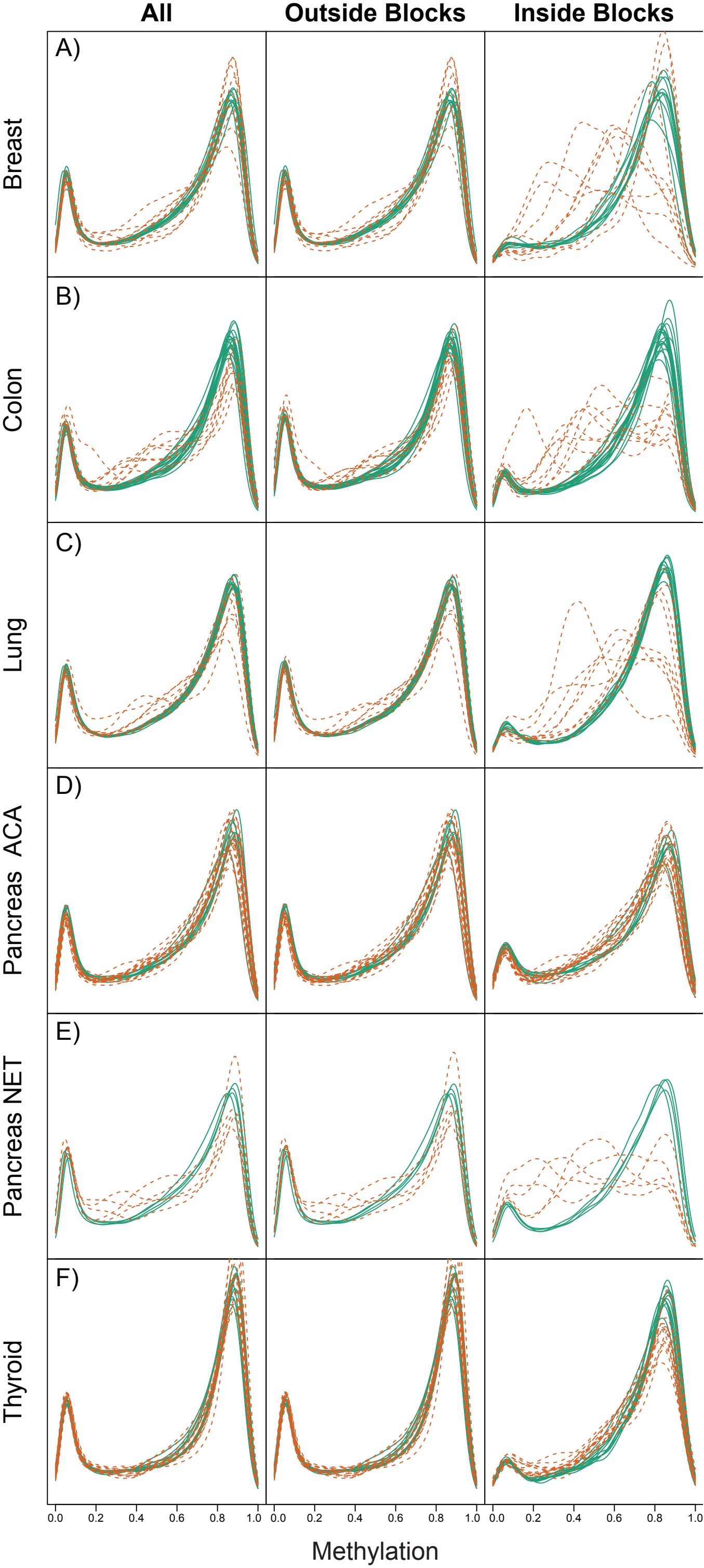

Supplement: Additional file 3: Figure S2. — Methylation density plot for normal (green solid lines) and cancer samples (orange dotted lines) for (left) all CpGs, (middle) CpGs outside of blocks and (right) CpGs inside blocks. A, B, C, D, E, and F correspond to breast, colon, lung, pancreas adenocarcinoma, pancreas neuroendocrine tumor, and thyroid, respectively. [file 13073_2014_61_MOESM3_ESM.pdf]

**A**

colon

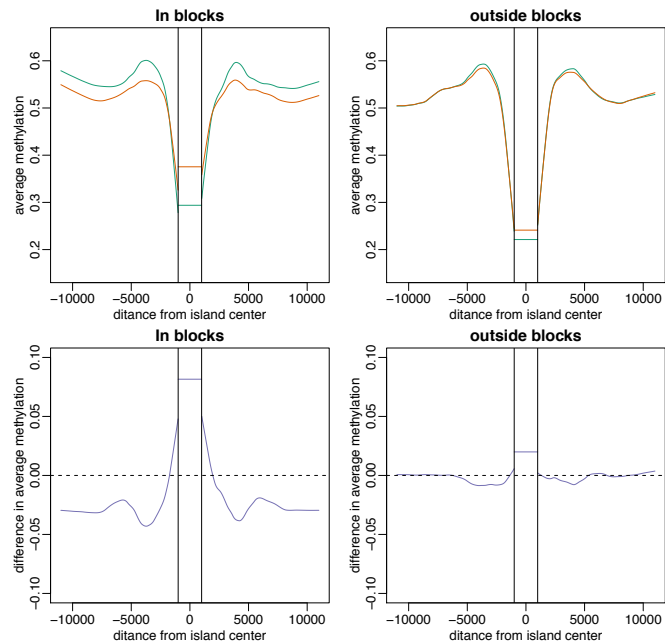

# B

lung

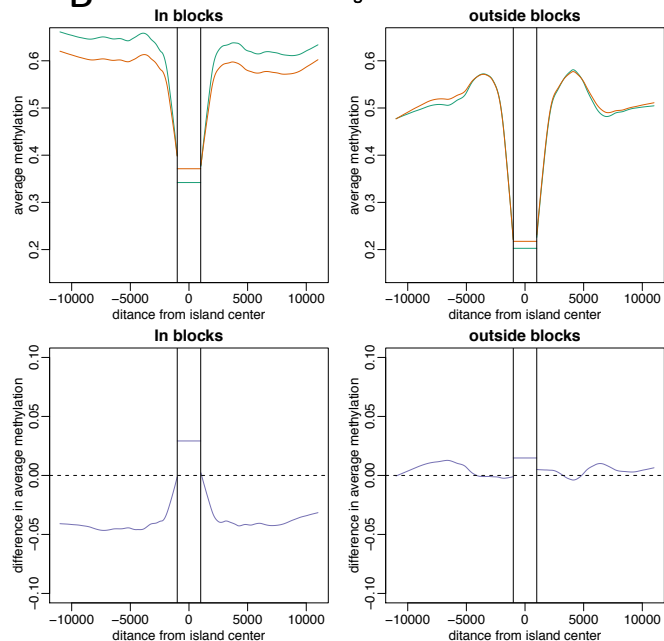

C

pancreas

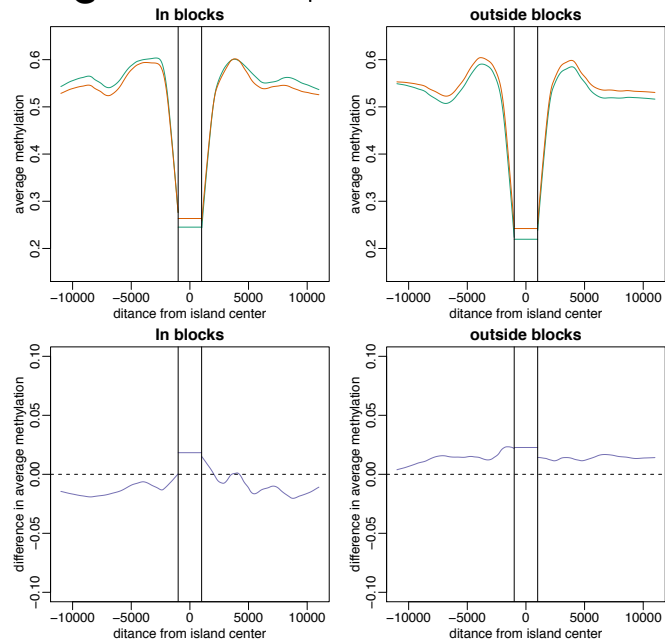

D

thyroid

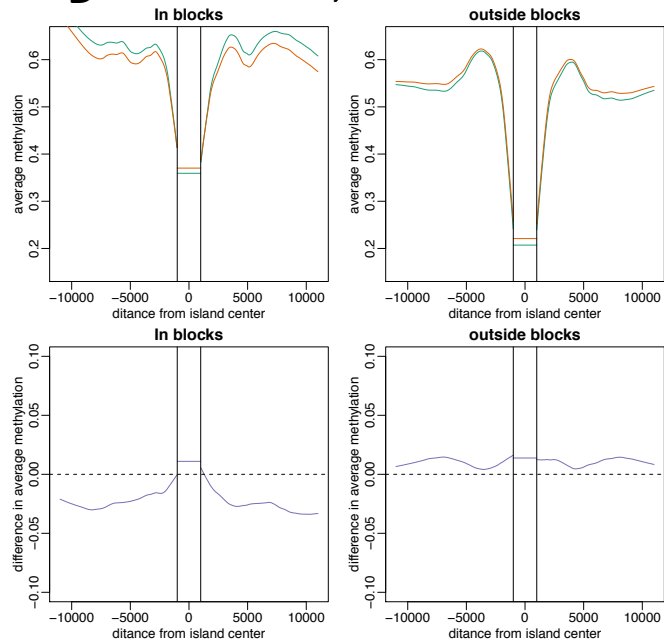

Supplement: Additional file 4: Figure S3. — As Figure 3 but for (A) colon, (B) lung, (C) pancreas, and (D) thyroid. [file 13073_2014_61_MOESM4_ESM.pdf]

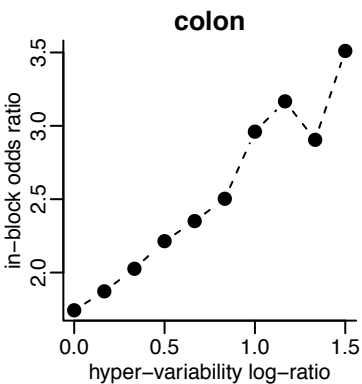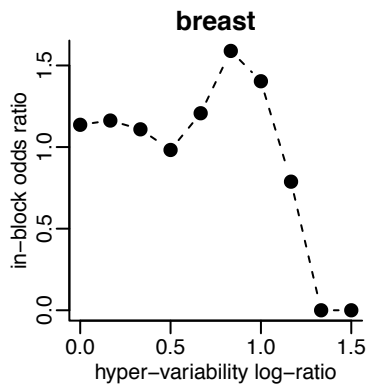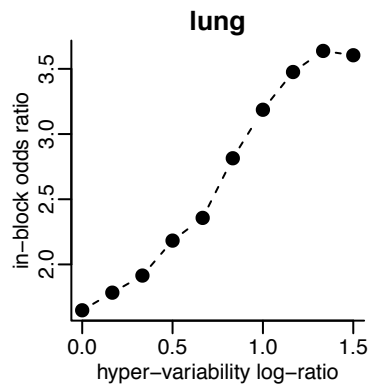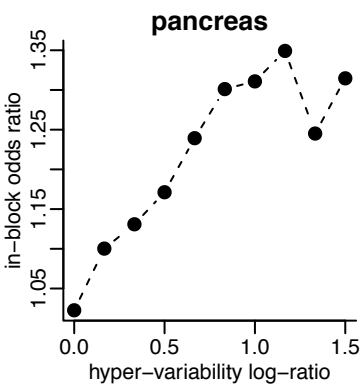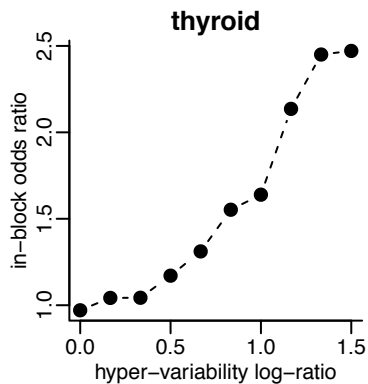

Supplement: Additional file 5: Figure S4. — Cancer gene expression hyper-variability is enriched in large hypomethylation domains in most solid tumor types. We obtained publicly available gene expression microarray data for each of the five tissues profiled and computed the log ratio of observed variability to expected variability (conditioned on mean expression) for each gene in each of the five cancer types. We plot the odds ratio of a gene's TSS being located within a detected hypomethylation block in each tissue given that observed to expected variability (OEV) is above increasing thresholds. We observed consistent increase in the odds ratio as the OEV threshold increases suggesting that gene expression hyper-variability is enriched in each tissue’s hypomethylation blocks. [file 13073_2014_61_MOESM5_ESM.pdf]
